# Awareness of radiation risks by medical students & referrers requesting radiological examinations in the North of Scotland: an audit

**DOI:** 10.1186/s12909-024-05461-8

**Published:** 2024-08-01

**Authors:** Shannon Mellis, Yuxuan Zhang, Dympna McAteer

**Affiliations:** 1 Medical MBChB Graduate 2023, University of Aberdeen School of Medicine and Dentistry, Polwarth Building, Foresterhill Rd, Aberdeen, AB25 2ZD UK; 2 ST3 Clinical Radiology, Aberdeen Royal Infirmary, Foresterhill Health Campus, Foresterhill Rd, Aberdeen, AB25 2ZN UK; 3 Consultant Radiologist, Aberdeen Royal Infirmary, Foresterhill Health Campus, Foresterhill Rd, Aberdeen, AB25 2ZN UK

**Keywords:** Ionising radiation, Radiology teaching, Medical training, NHS Scotland

## Abstract

**Introduction:**

Radiological imaging has played an important role in diagnostic medicine for over a century, though it is known to contribute to dermatological conditions, cataracts, and cancer. The associated risk of harm has led to the introduction of protective regulations around the world. Present-day NHS clinicians are increasingly requesting and relying on diagnostic imaging. Knowledge surrounding the radiation doses of common radiological investigations and the associated risks is imperative, and on a global level has been found to be inadequate. Consequently, there is a need for the formal inclusion of teaching within training programmes.

**Aims/objectives:**

This prospective audit aims to establish the knowledge of radiation doses and risks of common radiological investigations of both medical students and referrers within four NHS Health Boards based in the North of Scotland. It also seeks to establish prior teaching and the preference for further educational interventions.

**Audit standard:**

Referrers should have adequate knowledge of radiation doses and the risks associated with common radiological investigations.

**Audit target:**

The standard should be achieved by 90% of referrers.

**Methods:**

A 19-question online survey was devised to include subjective and objective questions on ionising radiation awareness, education preference, and respondent demographics, based on RCR (Royal College of Radiologists) audit criteria and previous studies. Data collection was conducted between the 22/02/23 to the 22/03/2023 and the questionnaire was distributed to senior medical students and radiological referrers of different grades within NHS Grampian, NHS Highland, NHS Shetland, and NHS Orkney. A descriptive analysis of the data was undertaken using Microsoft Excel Version 16.71.

**Results:**

Two hundred eight questionnaires were completed. 22.11% (*n* = 46) of the sample population had received no prior teaching on the topic of ionising radiation. Over half of the respondents (51.92%, *n* = 108) rated the importance of radiation risks as either important or extremely important, with 69.71% (*n* = 145) of participants rating their perceived knowledge as limited or average. Most correctly identified that a CT scan (*n* = 203), PET-CT scan (*n* = 199) and a chest x-ray (*n* = 196) exposed patients to ionising radiation. A small proportion of the participants incorrectly thought that an MRI scan (*n* = 21) and an ultrasound scan (*n* = 2) involved ionising radiation. The results obtained failed to meet the RCR audit target, which states that 90% of doctors should be aware of common radiological doses. It was observed that only 17.79% (*n* = 37) of survey respondents scored over 50% in the knowledge assessment, with the median knowledge score of the whole cohort being 2.5 out of 9 (27.78%).

Respondents who had prior teaching on the topic performed better those who had no prior teaching, with average scores of 3.19 (35.44%) and 2.04 (22.67%) respectively. Senior clinicians performed better when compared to junior clinicians and medical students.

**Conclusion & future recommendations:**

This audit found that the knowledge of radiation risks within the North of Scotland in the selected sample population was insufficient across all levels of the clinical team. Further, continuous education around the topic and future audit opportunities may help to optimise knowledge and training.

**Supplementary Information:**

The online version contains supplementary material available at 10.1186/s12909-024-05461-8.

## Introduction

### Ionising radiation: a history

Radiological imaging has played an important role in diagnostic medicine for over a century; since the discovery of the X-ray in 1895 by Professor Wilhelm Roentgen [1]. During the initial period of discovery, the dangers of ionising radiation were not fully understood. However, as time progressed, it became clear that human exposure to high doses of radiation could contribute to the development of dermatological conditions, cataracts, and cancer [2].

The harmful effects of ionising radiation can be classed as either deterministic or stochastic. Deterministic effects describe the direct tissue damage incurred by high doses of radiation, for example relating to skin and ocular damage. Stochastic effects describe those linked to cancer, and are outlined by the linear-no-threshold model. This details that the probability of genetic damage and carcinogenesis increases proportionally with increasing radiation exposure, though there is no threshold limit and the severity remains dose-independent [2].

The risk of harm with ionising radiation led to the introduction of protective regulations worldwide [3]. This practice of radiation protection is something that has evolved over time [4], and currently within the UK, this is provided by The Ionising Radiation (Medical Exposure) Regulations IR (ME)R (2017) legislation [5]. The guidance exists to protect both patients and employees from the dangers posed by ionising radiation within the medical environment. Guidance states that radiation exposure to patients must be as low as reasonably practicable (ALARP) and that the overall benefit of the radiological investigation must outweigh the risks from radiation [5].

In the present-day NHS, clinicians are requesting and relying on diagnostic imaging more than ever before. Comparatively speaking; 5.6 million computed tomography (CT) scans were carried out within NHS England in 2018–19, an increase from 3.3 million in 2012–13 [6]. This increased utilisation of CT imaging over the last decade can also be observed in other developed countries [7]. Newer imaging techniques such as positron emission tomography–computed tomography (PET-CT) and single-photon emission computed tomography (SPECT-CT), which expose patients to even higher doses of radiation, have also contributed to an increased dose burden [8].

### The current evidence contextualising risk

The increased utilisation of radiological investigations may be a cause for concern, as the radiation risks posed from modalities employing high doses of ionising radiation such as CT imaging are significant.

It is estimated that the lifetime risk of cancer from one CT abdomen or pelvis investigation is 1 in 2000 in adults [9], with potential for a cumulative effect after multiple scans [10]. Recent estimates in the literature state that 6 in 1000 cancers within the UK can be linked to ionising radiation [11], with the most common types of malignancy attributed to radiation reported as leukaemia, breast, thyroid, brain, and lung cancers [12].

The cancer risk is more significant in the paediatric population, in whom it is known that the lifetime risk of fatal cancer is proportionally higher, due to both extended life expectancy and increased cell radiosensitivity [13–16]. Female populations also have a greater susceptibility to carcinogenic effects, particularly with chest irradiation [17, 18].

However, the magnitude of the potential cancer risk from exposure to ionising radiation is not well understood. Most of the data regarding the cancer risk of radiation has come from long term studies of the Hiroshima and Nagasaki atomic bomb victims following World War II, something which is not directly comparable to the radiation that patients are exposed to in clinical practice [8, 19]. The atomic blasts contained neutrons and high energy gamma rays whereas medical imaging uses x-rays and low energy gamma rays [19]. The populations were exposed to a high full body exposure of radiation, and in clinical practice, the exposure during imaging is generally confined to a specific area [19].

There is also the contribution of genetic and environmental factors to carcinogenesis, which differs between individuals [20]. Advancements in CT technology has also led to improvements in protection, image efficiency and quality, and radiation dose optimisation [21, 22].

To conclude, further research in the area is required, to be able to accurately quantify the cancer risks posed by ionising radiation.

### Medical professionals’ knowledge of radiation risks

Despite the scientific uncertainties in quantifying low dose radiation risks, it is currently assumed that at low doses some level of risk remains. Therefore, a sufficient knowledge base is necessary in clinical practice to minimise this risk, and to ensure that investigations involving ionising radiation are justified, optimised, and patient centred [10]. An understanding of the topic is also important to educate patients and gain informed consent for imaging studies. Given that the number of investigations using ionising radiation has risen substantially in the past decade, the need for a sound knowledge base on the topic is more imperative than ever before [23].

The literature highlights, on a global level, that the knowledge possessed by medical students and clinicians regarding important concepts of ionising radiation is inadequate [23–52]. This has been demonstrated in a range of clinical settings, with studies taking place in medical schools, emergency departments, and clinical wards. Understanding has been noted to be particularly poor within cohorts of medical students and junior clinicians, though an improvement of knowledge with increasing experience and seniority is observed in some studies [28, 40, 42, 48].

Many studies suggest a unanimous need for the formal inclusion of teaching of radiation legislation and doses, within both the medical curriculum and work-based environments [24–32, 38–40, 43–46]. Some studies and audits within this subject area have shown an improvement in knowledge after a brief educational intervention [28, 32–34, 42, 48, 50, 51].

### Project aims and objectives

#### Aim

This prospective audit aims to establish the knowledge of radiation doses and risks of common radiological investigations of both senior medical students and referrers within NHS Health Boards based in the North of Scotland.

#### Objectives

- To determine the knowledge of radiation doses and risks of common radiological investigations
- To establish prior teaching on the topic of ionising radiation
- To establish the preferred modality of teaching for further educational intervention

#### Audit standards

The standards for this audit are based on standards set by The Royal College of Radiologists (RCR), which outline that 90% of doctors should be aware of the radiation doses associated with common radiological investigations [32, 53].

#### Hypothesis

Knowledge regarding radiation doses and risks of common radiological investigations is not expected to achieve the audit standards, in view of existing world literature. It is anticipated that there will be an increased knowledge level with increased experience of clinicians.

## Methodology

### Literature review

A literature search was carried out using Medline, Embase, and The Cochrane Library using the search terms attached in Appendix 1. Relevant publications, audits, and abstracts were identified for consideration. The RCR, (IR(ME)R) 2017, iRefer, and other relevant guidelines were also consulted.

### Questionnaire design

A 19-question online survey was devised to include questions on important concepts of ionising radiation, which can be observed with the annotated answers in Appendix 2. The questions were based on clinical practice, previous studies, and RCR audit criteria [24–29, 32, 53]. The format of the questionnaire included both multiple choice and written response questions. It covered demographic information, respondents’ prior knowledge of the subject, and any previous teaching they had received. The respondent was then asked to rate their perceived knowledge and importance of the subject area on a 7-point Likert scale. This was then followed by knowledge-based questions on radiation doses of commonly requested radiological examinations, the attributed lifetime risk of cancer of these examinations, and a question on the patient groups most sensitive to ionising radiation. A chest x-ray (CXR) was used as a unit dose for the radiation knowledge questions as it was felt unreasonable to expect participants to know the effective dose of radiation in scientific units such as mSv. Answers were based on radiation doses extracted from the UK Government website [9]. The questionnaire then provided an opportunity for additional comments and views on future teaching opportunities.

Prior to release, the questions were reviewed by a consultant radiologist and a radiology trainee. It was transferred onto Microsoft Forms for distribution and a pilot study was carried out amongst a small group of doctors to ensure that the questions were understandable, coherent, and that the relevant data was obtained.

### Data collection

Data collection was conducted between the 22/02/23 to the 22/03/2023. The questionnaire was distributed to medical students and radiological referrers of different grades within four different health boards based in the North of Scotland: NHS Grampian, NHS Highland, NHS Shetland, and NHS Orkney. In the smaller health boards, the survey was distributed to all doctors, advanced nurse practitioners (ANPs), and physician associates (PAs) via staff emailing lists. In the larger teaching hospitals smaller departments were invited to take part via email and QR codes. The University of Aberdeen Medical School distributed the survey via email to all final and penultimate year medical students.

### Ethical considerations

Consent was obtained from participants prior to the completion of the questionnaire to enable the use of collected data. Approval from the North of Scotland Research Ethics Service or NHS Grampian Research and Development Department was not deemed necessary for this study. The audit was registered with the Quality Improvement & Assurance Team based within NHS Grampian.

### Data analysis

A descriptive analysis of the data was undertaken using Microsoft Excel Version 16.71. The results for comparison of demographic data and knowledge scores were presented using the median and interquartile range, due to the positive skew of the knowledge assessment results.

## Results

### Respondent demographics

Two hundred eight questionnaires were completed in total, with demographic information summarised in Table 1. The response rate from penultimate and final year medical students was 14.3% (*n* = 33) and 27.1% (*n* = 51) respectively. The response rate from other healthcare professionals cannot be accurately reported due to limitations associated with the distribution processes. Participants selecting “Other” as health board included: NHS Western Isles, NHS Greater Glasgow and Clyde and NHS Dumfries and Galloway. Participants selecting “Other” as grade included: staff nurses, a paramedic and locum doctors.

**Table 1:** Demographic information of respondents

Characteristics	Number (n)	Percentage of Sample (%)
**Health Board**		
NHS Grampian	**139**	**66.8**
NHS Highland	**49**	**23.6**
NHS Shetland	**12**	**5.8**
NHS Orkney	**3**	**1.4**
Other	**5**	**2.4**
**Setting**		
Primary Care	**34**	**16.3**
Secondary Care	**174**	**83.7**
**Grade**		
Penultimate Year Medical Student	**33**	**15.9**
Final Year Medical Student	**51**	**24.5**
Junior Doctor (FY1/FY2/Junior CDF)	**33**	**15.9**
Junior Middle Grade Doctor (CT/IMT/GPST/ST1-2)	**14**	**6.7**
Senior Middle Grade (ST3 +)	**17**	**8.2**
GP	**13**	**6.3**
Speciality Doctors (SAS)	**6**	**2.9**
Consultant	**18**	**8.7**
Advanced Nurse Practitioner	**12**	**5.8**
Physician Associate	**6**	**2.9**
Other	**5**	**2.4**

**Fig. 1:**
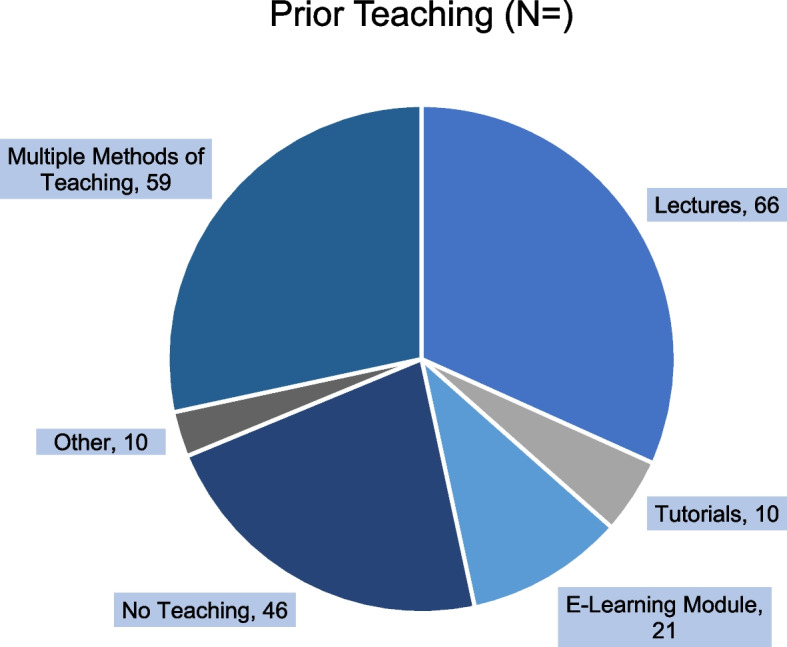
Prior teaching modalities that respondents received on the topic of ionising radiation

Figure 1 displays the prior teaching that respondents had on the topic of ionising radiation, with 22.11% (*n* = 46) stating they had received no prior teaching. Respondents were able to select multiple answers in this question as observed.

### Awareness of radiation risks

**Fig. 2:**
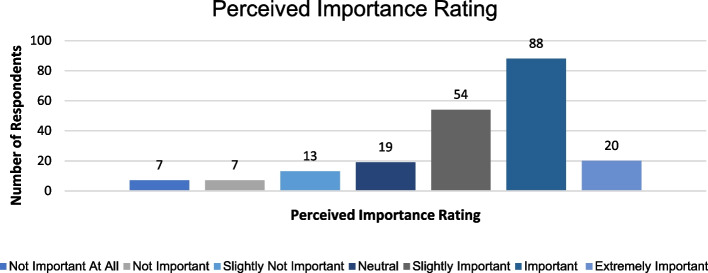
Perceived importance ratings of the knowledge of radiation risks of common radiological investigations

**Fig. 3:**
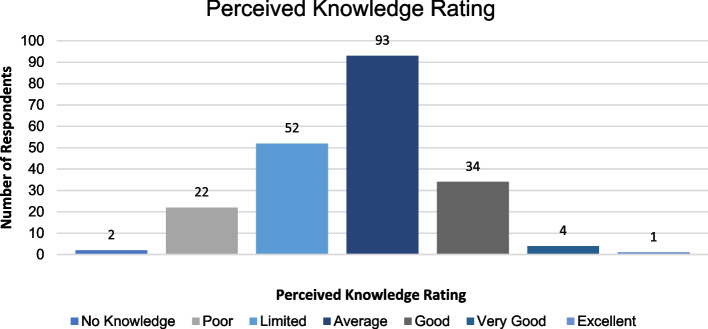
Perceived knowledge ratings of the radiation risks of common radiological investigations

Figures 2 and 3 represent the respondents’ perceived importance and perceived knowledge of radiation risks, which participants were asked to rate on a 7-point Likert scale.

51.92% (*n* = 108) of the sample size rated the importance of radiation risks as either important or extremely important. This contrasts with the perceived knowledge scores, in which 69.71% (*n* = 145) of participants rated their knowledge as limited or average.

Respondents were then asked questions to assess knowledge. The first part of the questionnaire determined whether participants knew which radiological imaging techniques involved ionising radiation. Most correctly identified that a CT scan (*n* = 203), PET-CT scan (*n* = 199), and chest x-ray (*n* = 196) exposed patients to ionising radiation. A lower number of respondents correctly identified that a mammogram (*n* = 150) or angiogram investigation (*n* = 160) involved ionising radiation. A small proportion of the participants incorrectly thought that an MRI scan (*n* = 21) and an ultrasound scan (*n* = 2) involved ionising radiation.

Tables 2 and 3 summarise the results of further knowledge assessment questions. Participants were asked what the chest x-ray radiation dose equivalent was for each of the imaging investigations and then what the lifetime attributed risk of cancer was on exposure.

**Table 2:** Distribution of results for equivalent number of chest x-rays of common radiological investigations

Radiological Investigation	Effective Radiation Dose as Equivalent Number of Chest X-rays	% of Correct Responses	% Underestimated the Radiation Dose	% Overestimated the Radiation Dose	% Answered Don’t Know
Abdominal X-ray	**35**	**14.42 (*n* = 30)**	**46.63 (*n* = 97)**	**11.06 (*n* = 23)**	**27.88 (*n* = 58)**
CT Head	**100**	**26.92 (*n* = 56)**	**36.54 (*n* = 76)**	**6.25 (*n* = 13)**	**30.29 (*n* = 63)**
CT Chest	**400**	**22.60 (*n* = 47)**	**21.15 (*n* = 44)**	**23.56(*n* = 49)**	**32.69 (*n* = 68)**
CT Abdo/Pelvis	**500**	**19.71 (*n* = 41)**	**45.67 (*n* = 95)**	**N/A – no higher option**	**34.62 (*n* = 72)**

**Table 3:** Distribution of results for additional lifetime risk of fatal and non-fatal cancer from the following investigations

Radiological Investigation	Additional Lifetime Risk of Cancer	% of Correct Responses	% Underestimated the Cancer Risk or No risk	% Overestimated the Cancer risk	% Answered Don’t Know
CT Head	**1 in 10,000**	**36.06(*n* = 75)**	**2.88 (*n* = 6)**	**22.60 (*n* = 47)**	**38.46(*n* = 80)**
CT Chest	**1 in 2500**	**23.56(*n* = 49)**	**19.23(*n* = 40)**	**16.35(*n* = 34)**	**40.87(*n* = 85)**
CT Abdo/Pelvis	**1 in 2000**	**18.74(*n* = 39)**	**26.44(*n* = 55)**	**13.94(*n* = 29)**	**40.87(*n* = 85)**

### The knowledge score

Questions assessing knowledge were added up to give a score out of 9, with an incorrect answer or “I don’t know” given zero marks and a correct answer given one mark. Questions with multiple correct answers were given one mark if answered fully correct or half a mark if over half of the selection was answered correctly as displayed in Appendix 2. No negative marking was used.

**Fig. 4:**
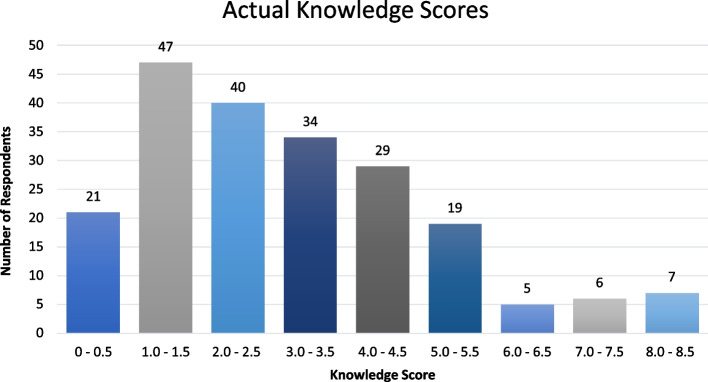
Actual knowledge scores of respondents (Scored out of 9)

Figure 4 displays the actual knowledge scores of the respondents, with a higher score indicating greater knowledge on the topic.

The median knowledge score for the entire cohort was 2.5 out of 9 (27.78%), and scores ranged from 0 to 8.5. It was shown that 82.21% (*n* = 171) of the sample scored 50% or less on the knowledge assessment.

It was observed that respondents who had received teaching on the topic of ionising radiation performed better in the knowledge assessment than those who had no prior teaching, with average scores of 3.19 (35.44%) and 2.04 (22.67%) respectively. Three of the knowledge scores were excluded from this calculation as respondents could not remember if they had received prior teaching.

**Fig. 5:**
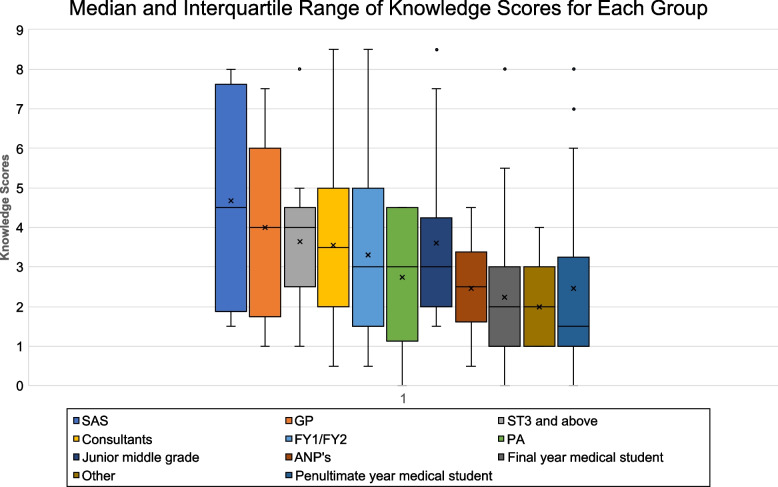
Median and interquartile range of knowledge scores for each group

The median and interquartile range of the different subgroups of respondents is displayed in Fig. 5 and Table 4 to allow comparison.

**Table 4:** Median and interquartile range of knowledge scores for each group

Value	SAS	GP	ST3 and above	Consultants	FY1/FY2/CDF	PA	Junior middle grade	ANP's	Final year medical student	Other	Penultimate year medical student
Minimum	1.5	1	1	0.5	0.5	0	1.5	0.5	0	0	0
Q1	2.5	2	3	2	1.5	1.625	2.3	1.9	1	1	1
Median	4.5	4	4	3.5	3	3	3	2.5	2	2	1.5
Q3	6.88	6	4.5	4.75	5	4.375	3.9	3.1	3	3.5	3
Maximum	8	7.5	8	8.5	8.5	4.5	8.5	4.5	8	8	8
IQR	4.38	4	1.5	2.75	3.5	2.75	1.6	1.3	2	2.5	2

As shown in Fig. 5, the group attaining the highest median knowledge score was the speciality doctor group (SAS), with a score of 4.5 (50.00%). However, the knowledge scores within this group showed greater disparity when compared to other groups.

Following this, the GP, and senior middle grade doctor (ST3 and above) cohorts performed the best, with joint median knowledge scores of 4 (44.44%). The GP cohort had a wider range of knowledge scores, whereas the senior middle grade doctors and consultants showed greater similarities with knowledge scores.

The poorest performing cohorts were the final and penultimate year medical students, who achieved median knowledge scores of 2 (22.22%) and 1.5 (16.67%) respectively. Respondent scores showed greater congruence within this group, as indicated by a smaller interquartile range. This group were also found to be the most likely to answer knowledge questions with “I don’t know”. The “other” group also scored poorly with a median knowledge score of 2 (22.22%).

**Fig. 6:**
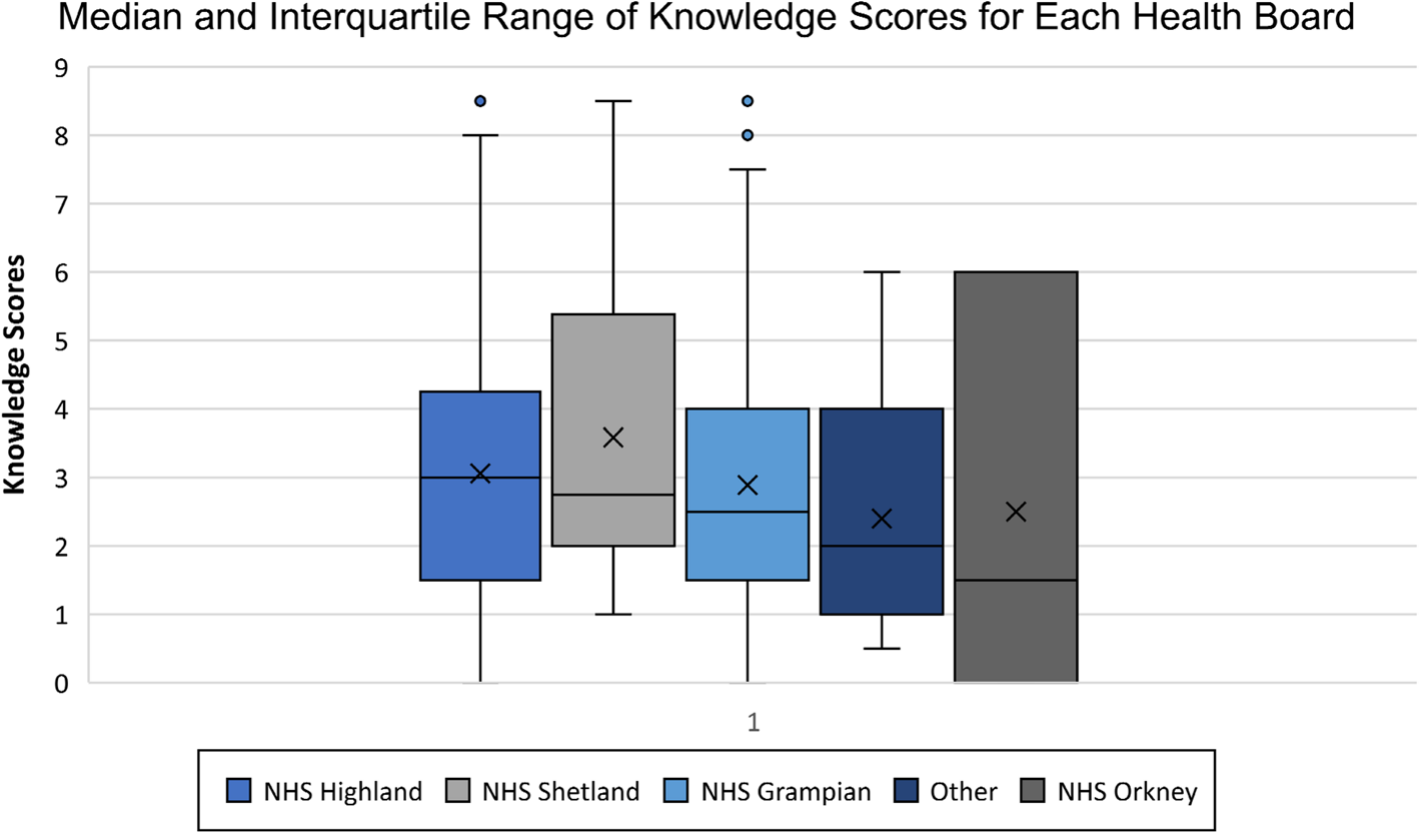
Median and interquartile range of knowledge scores for each health board

The median and interquartile range of the different health boards is displayed in Fig. 6 and Table 5 to allow comparison.

**Table 5:** Median and interquartile range of knowledge scores for each health board

Values	NHS Grampian	NHS Highland	NHS Shetland	NHS Orkney	Other
Minimum	0	0	1	0	0.5
Q1	1.5	1.5	2	0.75	1.5
Median	2.5	3	2.75	1.5	2
Q3	4	4	5.125	3.75	2
Maximum	8.5	8.5	8.5	6	6
IQR	2.5	2.5	3.125	3	0.5

The median knowledge scores for each health board were similar, though within each group there was a wide spread of knowledge scores.

The health board attaining the highest median knowledge score was NHS Highland, with a median knowledge score of 3 (33.33%). The results for this health board followed a normal distribution, with most results clustered around the median, with some outliers.

The health board with the lowest median knowledge score was NHS Orkney with a score of 1.5 (16.67%), though it is important to note the smaller size of this sample group (*n* = 3).

The last knowledge-based question covered sensitivity to radiation, in which 78.85% (*n* = 164) of respondents were aware of the higher risk of radiation to children, and 43.27% (*n* = 90) were aware of a higher risk to females. 9.13% (*n* = 19) of respondents felt that there were equal risks to the whole population.

### Further teaching

**Fig. 7:**
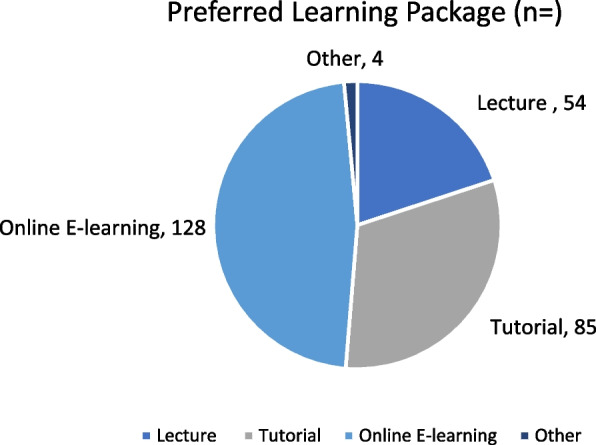
Preferred learning package results

Most respondents felt that further teaching on the topic of ionising radiation would be beneficial (85.57%,*n* = 178). The type of learning package that participants would prefer is displayed in Fig. 7.

The “Other” comments included suggestions such as a summary poster to be displayed within ward areas to aid knowledge.

There was then an opportunity for participants to leave further comments at the end of the questionnaire, these are detailed in Appendix 3.

## Discussion

### Knowledge of radiation risks

The aim of this study was to establish the knowledge of radiation doses and risks of common radiological investigations of both medical students and referrers within NHS health boards based in the North of Scotland. The key finding in this audit was that the overall knowledge of respondents on this topic was poor.

The results obtained fail to meet the RCR audit target, which states that 90% of doctors should be aware of common radiological doses. It was observed that only 17.79% (*n*= 37) of survey respondents scored over 50% in the knowledge assessment, with the median knowledge score of the whole cohort being 2.5 out of 9 (27.78%). The results support the hypothesis and align with the results of other global studies surveying similar concepts [24–52].

From the self-rating scale, most participants felt that knowledge of radiation risks was important (*n* = 88, 42.31%), but most also felt they only had average knowledge (*n* = 93, 44.71%). This indicates that the respondents may lack confidence in the topic, but recognise it is an important aspect of clinical practice.

Within this study it was found that senior clinicians did perform better than junior colleagues, with the groups obtaining the highest median knowledge scores being attained by the speciality doctors (SAS), senior middle grade doctors, GPs, and consultants. The number of respondents in the SAS subgroup was small (*n*= 6), so it was felt this may have influenced the results for this cohort. However, the remaining senior doctor subgroups were larger, and the individual results were mostly found to be more congruent, strengthening the observation that senior clinicians have a higher knowledge base than junior colleagues. The increased knowledge base with increased level of experience resonates with other similar studies [28, 40, 42, 48].

Conversely, another study carried out in Australia by Brown et al. [29] demonstrated a statistically significant inverse relationship between years of experience and knowledge of radiation risks, suggesting that knowledge within the senior clinician cohort was poorer. Though Brown’s study had twice the number of less experienced doctors which might have influenced this finding.

The poorest results in this study came from the two largest cohorts; the penultimate and final year medical students, with median knowledge scores of 1.5 (16.67%) and 2 (22.22%) respectively. The “other” group also scored poorly with a median knowledge score of 2 (22.22%), though this group had only five participants from various non-medical backgrounds.

When comparing to other studies which focus exclusively on medical students, the knowledge assessment results in this study were found to be significantly poorer, with a Norwegian study by Kada et al [24], observing average knowledge scores of final year medical students to be 35.55%. A similar questionnaire design was used, though there was approximately double the number of students in the Norwegian study, with a higher proportion reporting prior radiation teaching.

O’Sullivan et al [28] studied 670 medical students in Ireland and found that knowledge improved with years of experience, though overall knowledge remained poor. In the studied universities, O’Sullivan states that final year students participated in “intensive clinical radiology teaching” which may account for this improvement. The findings were similar to this project, in that final year medical students performed better than the penultimate year students, though there is no formal teaching on radiation protection between these years in Aberdeen which can account for this. The poor results portrayed in this audit could be due to this lack of teaching within the university’s curriculum.

When the results of the whole cohort (*n* = 208) are compared to the literature, several studies demonstrated superior radiation knowledge amongst participants. Uri [25] published a study within England assessing knowledge of radiation protection of referrers within three hospitals, where almost double the respondents correctly identified radiation doses associated with a number of CT investigations, when compared to this audit. For example, 44% of study respondents were able to identify the correct radiation dose of a CT head, compared to 26.92% in this audit. The demographics of Uri’s study respondents contained a higher ratio of senior doctors, and medical students were not included, which may account for greater knowledge. Overall, they still found a large proportion of referrers underestimated the radiation risks, a finding replicated in this audit.

In Northern Ireland, Soye et al. [26] published that 155 doctors scored an average of 39% in their radiation knowledge questionnaire. In comparison, the results from this audit were found to be inferior, with the average knowledge score for the whole cohort being 32.78%. The Soye et al. study had comparatively more senior clinicians, with 93 of the 153 respondents being consultants, which might have contributed to their improved scores.

Conversely, other studies demonstrated inferior radiation knowledge when compared to this audit. Zhou et al. [27] carried out a similar study in Australia which gained responses from 331 interns and medical students. They found that 89.3% and 88.9% of participants underestimated the radiation dose of abdominal x-rays and abdominal CT scans respectively. The study does not appear to give information on the breakdown of participants medical grade, making it difficult to further interpret results.

It was encouraging that most respondents within this audit were able to identify that magnetic resonance imaging (MRI) (90%) and ultrasound scans (USS) (99%) do not involve ionising radiation. This was an improvement from the rate of awareness in multiple other studies [24, 25, 27, 38, 40, 46]. This should be considered core medical knowledge as these investigations may be appropriate alternatives in a patient’s journey to prevent unnecessary exposure to radiation.

Faggioni et al [39] and Borgen et al [54] demonstrated that radiology residents and radiology students had significantly better knowledge of radiation and the associated guidelines than clinicians, which is to be expected with specialist training. Certainly, one respondent stated in the further comments section in Appendix 3, “*we expect the radiologists to advise us*”. This may be true of many clinicians working within different specialities, medicine already requires an extensive knowledge base, and perhaps it is not realistic to expect clinicians to memorise the finer details. Though it is important for them to understand common investigations and risks, especially those routinely requested within their clinical area.

Overall, this audit showed that knowledge of radiation could be improved upon. As discussed in another study by Ramanthan et al. [52] where radiation knowledge was found to be poor, if approximated radiation doses are not known, then clinicians might have a lower threshold for referral. This may lead to unwarranted or inappropriate referrals, particularly in cases where the benefits of investigating do not clearly outweigh the risks.

### Consent of the patient

Informed consent of the patient is an important consideration in the practice of medicine and the General Medical Council (GMC) states that *“shared decision making and consent are fundamental to good medical practice”* [55].

Without the appropriate knowledge to convey the risks and benefits of radiation to the patient, then it is unlikely that patients can make a fully informed decision. This study showed poor knowledge of radiation risks, which may lead to the assumption that details surrounding the imaging examinations are not being thoroughly discussed with the patient. The (IR(ME)R) 2017 [5] guidance states that prior to radiation exposure patients should be informed of the risks and benefits. This should at first instance, lie with the referrer. However, there may be reasons other than lack of knowledge that prevent clinicians from communicating the risks; such as time pressures, staff shortages, or other challenges within the working environment.

Within the literature it is shown that patients are often inadequately informed about the radiation exposure from radiological imaging, with one study stating that almost all of the patients receiving a CT scan were unaware of the risks [56]. Not only do patients have a right to be informed and involved in decisions regarding their care, it has been shown in the literature that the majority of patients want to understand the risks of radiation [57].

A study by Goske et al. [58] suggested the use of education tools for patients to give further information and contribute to informed consent, whilst also relieving any anxieties that patients or guardians may have. This resonated with a comment left by one of the respondents who stated “*You need to have significant thought about patient information in decision making, patients are frequently subjected to ionising radiation with zero consent, zero knowledge of the risks and decisions made by junior medical staff who have no experience or ability to consider broader clinical risks. I think the most beneficial outcome would be to have patient information leaflets on the wards explaining these risks*”. This may be a suitable suggestion to support the responsibilities of the referrer and ensure that the patient is aware of both the risks and benefits of imaging.

### Appropriateness of Imaging

Medical imaging has a crucial and, at times, life-saving role in the patient journey. In major trauma and emergency cases, CT imaging is superior to other modalities such as MRI, due to the rapid generation of information [59]. This is despite it delivering a significant radiation dose and the associated increased cancer risk. This sentiment was echoed by one of the audit respondents who commented “The problem is always balanced decision making. The issue of a 1/2500 cancer is not a priority when dealing with situations that have a 25% 30-day mortality risk.”

In the UK, imaging referrals are justified by a radiology specialist, a practitioner, to ensure they comply with published guidance. A national audit covering 88 radiology departments found that most imaging was indicated following review by a radiology specialist – with the recommended standard of over 90% of imaging being met [7].

This shows that most medical imaging is carried out judiciously due to rigorous referral processes in place, but there have been some studies suggesting that almost a third of all imaging carried out is inappropriate or unjustified, particularly with regard to younger patients [60–62].

One respondent in the survey commented *“The default of surgical teams to get CT scans for ED patients needs to be addressed—what happened to appendicitis being a clinical diagnosis? The defensive medical world that we live in means that the number of CT heads done in ED under NICE guidelines is ridiculous.”* This highlights complexities with referral decisions, suggesting that some clinicians may feel that certain imaging requests are inappropriate, or may feel obliged to request examinations on the basis of satisfying guideline requirements.

The facts surrounding inappropriate referrals may be debated, but without a solid understanding of the radiation risks, doctors may be unable to fully weigh up the risks versus benefits of imaging investigations, to justify patient exposure, and limit doses of radiation. Current legislation maintains that no ionising radiation is free from risk and that doses should be ALARP. Other considerations which might help referrers decide may be Cochrane’s Law; “*Before you request a test, you should first ask yourself what you are going to do if the test is positive, then ask yourself what you are going to do if the test is negative. If the answer is the same, do not do the test”* [63].

Additional references that may also help to support doctors with radiology referrals include the RCR’s iRefer guidance, which details appropriate indications for each investigation [64]. Though it is shown in the literature that awareness of this useful resource is limited [33–35].

### Additional risks of investigations

When considering the radiation risks of imaging investigations, it is also important to consider additional risks, such as the discovery of incidental findings. One respondent mentioned this in the further comments section, stating *“I work in Old Age medicine—generally lifetime risks are less relevant at this stage! We do consider other risks of investigation though, such as risks of over investigation and incidental findings”.*The rate of pick-up of incidental findings has been referenced as 3 to 30% during research imaging, with higher rates occurring in chest and abdominal imaging [65]. This risk is important to consider, as imaging may detect an abnormality that would have never become symptomatic in the patient or caused any harm. Such findings can lead to a long road of investigations, some of which may be invasive in nature, which may not be in the patient’s best interests. It is important to consider this as part of the wider context when referring patients for imaging investigations.

### Educational interventions

It was observed during this audit that respondents who had received prior teaching on the topic of ionising radiation performed better. A large proportion of the survey participants (*n*= 178,85.57%) also reported that they would benefit from further teaching on the topic of ionising radiation. This suggests that a targeted educational intervention could make a difference to the knowledge base of clinicians. Improved teaching on radiation protection is encouraged following multiple studies in the literature [28, 32–34, 42, 48, 50, 51].

### Within the medical school cohort

Patient safety has been an important aspect of medical education for a number of years, with the GMC’s First Do No Harm [66] outlining the importance of its integration into the medical degree, of which radiation protection is a small component.

Implementation of radiation protection teaching within the medical curriculum has also been suggested by several additional bodies. It is one of the patient safety competencies recommended in The WHO Patient Safety Curriculum Guide for Medical Schools [67]. The RCR have also detailed specific recommendations for radiology teaching within the undergraduate curriculum [68]. This includes the outcome that medical graduates should be able to understand ionising radiation risk and be able to counsel patients on the risks and benefits of imaging investigations and procedures.

At the university of Aberdeen, radiology teaching is integrated throughout the five-year programme, but from a personal perspective more of an emphasis may need to be placed on radiation protection to improve the knowledge of medical students. This is supported by the medical student cohorts scoring poorest in the survey, and the vast majority of students selecting “I don’t know” in response to questions. To improve knowledge, future implementation of e-learning or small group tutorials may be of benefit, to help students learn the core principles of radiation protection. Studies have both supported the use of radiation online modules and teaching from radiologists as medical students reach their final years [28, 50].

### Within the clinical staff cohort

Education on the topic within the hospital is also important, to inform those that may not have had appropriate teaching and to reinforce or refresh teaching within those that have.

Some positive findings in the literature found that implementation of various educational resources within the hospital environment worked well to improve knowledge. One study found that a pop-up message on the electronic record such as “This examination is the equivalent of 400 CXRs; do you really need to do it?” increased awareness of the radiation dose amongst referrers [23]. Another method suggested that radiation doses and the related risks should be provided on imaging request forms [69]. Some of the previous audits carried out showed improvement within junior doctor cohorts with small group teaching, to emphasise important concepts of radiation protection [32–36].

However, some studies have reported that an educational intervention made no difference to knowledge, so it is imperative that the most appropriate method of teaching is selected [48, 70].

With advancements in care there may be more nurses and other allied healthcare professionals requesting ionising radiation examinations, which is already in place in some health boards – so education would need to be widespread to include non-medically qualified referrers [71].

As previously discussed, imaging referral guidelines exist –a resource known as iRefer—and is accessible to all within NHS Scotland on the NHS intranet. It contains the RCR’s specific guidelines to help inform referrers (namely GPs and non-specialist referrers) when imaging is indicated and provide knowledge of common radiation doses. Radiology guidelines have been found to be useful for both a clinician’s knowledge base and ensuring appropriateness of referrals. One study, referring to the use of radiology guidelines within primary care, has been shown to reduce the number of requested examinations by 20%, improving the appropriateness of referrals [72, 73]. It is important that awareness of these guidelines is promoted in clinical areas, to support doctors in their decision-making processes.

Overall, further education on the topic of ionising radiation would be of benefit to the surveyed cohort, and this is something which needs future consideration for implementation into the workforce.

### Strengths and limitations

#### Strengths of the audit

The sample size was sufficient and comparable (*n* = 208), if not larger, than other similar studies or audits, strengthening the generalisability of the results. There was an evenly distributed engagement from a wide variety of healthcare professionals of differing levels of experience, to allow valid comparison of the knowledge levels at different stages of training. The responses covered a large geographical area, and responses were obtained from each of the different health boards, strengthening the reproducibility of results within the medical population. Relative strengths of the methodology include that the questionnaire design was simple and quick to complete, and there was an ease of distribution via an emailed link or printed QR code.

#### Limitations of the audit

Due to difficulties in distributing the survey via mainstream mailing systems in the larger teaching hospitals the surveys were instead distributed to smaller departments on a voluntary basis, which introduced a sampling bias. There is also the contribution of self-selection bias, which is intrinsic to online questionnaire methods, in that data was only collected from those who decided to take part. This may have led to an inaccurate observation of the knowledge scores and impact the external validity of the project. For future studies it is important that distribution of the questionnaire is considered in early project planning, and methods are used to reduce the risk of selection bias. Though the risk of bias is somewhat mitigated by the large sample size achieved overall. It is important to note the smaller numbers of responses received from NHS Orkney and NHS Shetland, though it was felt this was still a comparative sample when comparing to the larger workforce present in NHS Grampian and NHS Highland. Another consideration is that due to the nature of the questionnaire, some people may have referred to external guidance when answering the knowledge questions, thereby affecting their knowledge score.

### Future recommendations & action plan

**Fig. 8:**
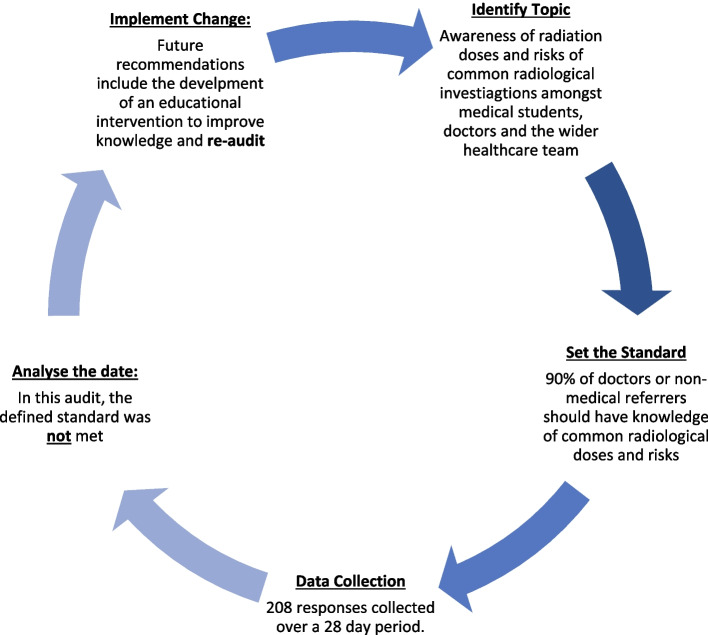
The audit cycle

Future recommendations would involve completing the audit cycle displayed in Fig. 8. It would be beneficial to educate staff on the results of this audit, and to introduce an educational package. The most popular learning modality was found to be an e-learning module, and this would allow for asynchronous learning opportunities.

It may also be of benefit to supplement new learning by distributing posters within clinical areas, to highlight the pertinent points of the use of ionising radiation. This could also serve to remind clinicians of the recommended referral guidelines—iRefer.

A re-audit should be undertaken to assess the knowledge base following an educational intervention, to show if an improvement in knowledge has occurred, and to consider if it is something that should be implemented; within the medical school curriculum or NHS induction process.

A future audit may also want to incorporate the specific department of work as part of the demographic information collected for analysis, to compare the knowledge base in departments requesting varying levels of radiological investigations. It may also be of interest to include a patient survey or interview, to cover the views of patients on the topic, in future audit endeavours.

## Conclusion

This audit found that the knowledge of radiation risks within the North of Scotland in the selected sample population was insufficient across all levels of the clinical team. While the value of medical imaging is undisputed, it is important that the risks of such referrals are considered in the decision-making process. Furthermore, continuous education around the topic and future audit opportunities may help to optimise knowledge. Improving conversations with patients around these investigations will also help to improve patient centred care and the decision-making process.

### Supplementary Information

**Supplementary Material 1.**

## Data Availability

The datasets used and/or analysed during the current study are available from the corresponding author on reasonable request.

## Abbreviations

ALARP: As low as reasonably practicable
AXR: Abdominal x-ray
CT: Computed tomography
CXR: Chest x-ray
GMC: General Medical Council
(IR(ME)R) 2017: The Ionising Radiation (Medical Exposure) Regulations IR (ME)R (2017) legislation
MRI: Magnetic resonance imaging
PET-CT: Positron emission tomography–computed tomography
RCR: The Royal College of Radiologists
SPECT-CT: Single-photon emission-computed tomography
WHO: World Health Organisation
