# Supplementary material for: Awareness of radiation risks by medical students & referrers requesting radiological examinations in the North of Scotland: an audit
Source: BMC Med Educ. 2024 Aug 1;24:830. doi: 10.1186/s12909-024-05461-8 (PMC11295379; doi:10.1186/s12909-024-05461-8)
Supplement: Supplementary file 1 — Supplementary Material 1. [file 12909_2024_5461_MOESM1_ESM.docx]

*Appendices*

# **8.0 Appendices**

## *8.1 Appendix 1: Search Terms*

**
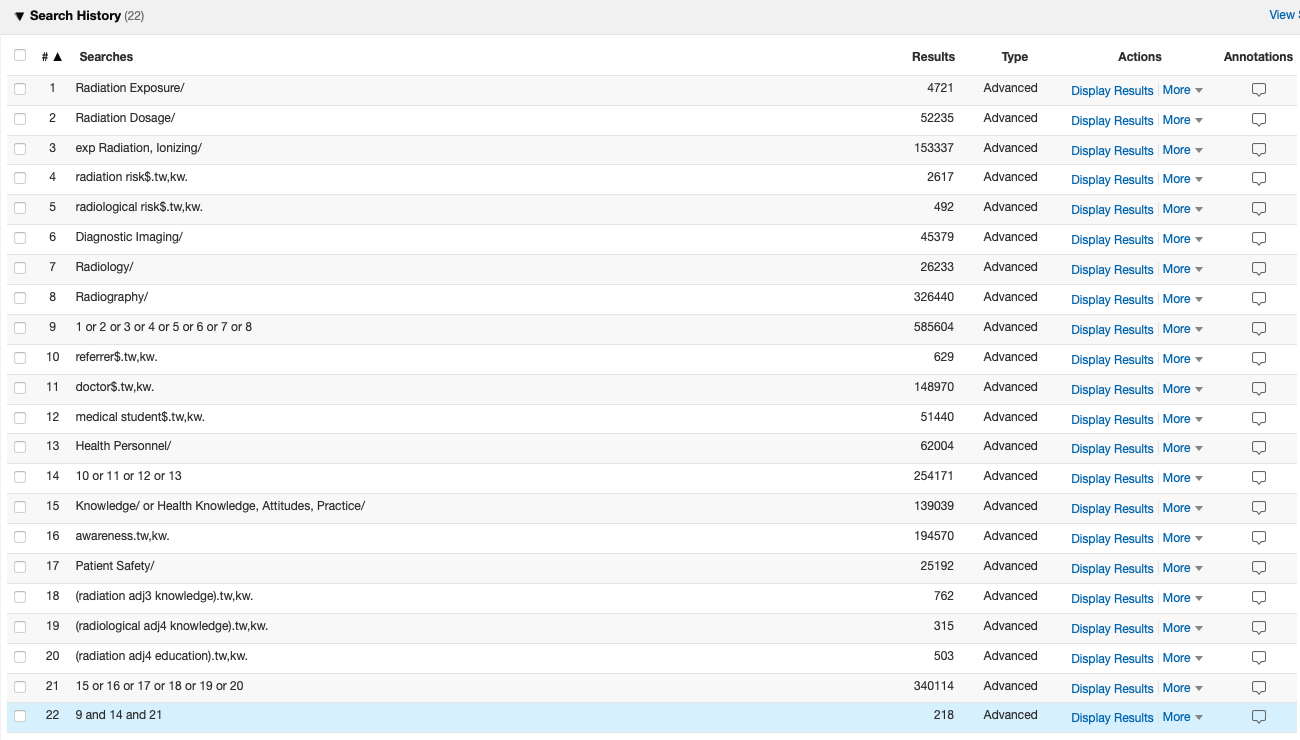
**

## *8.2 Appendix 2: The Questionnaire*

**Awareness of Radiation Risks**

**Awareness of Radiation Risks by Referrers Requesting Radiological Examinations by Shannon Mellis, Year 5 Medical Student, University of Aberdeen.**

*The aim of this questionnaire is to assess the knowledge surrounding radiation doses of commonly requested investigations in clinical practice. All results will be anonymised, and no identifiable information will be used in the results of this study. Data will remain secure and will be used within the constraints of this study. By completing and submitting the questionnaire you are consenting for your data to be used. Any responses submitted will be greatly appreciated. This questionnaire is part of a year five elective project for the University of Aberdeen MBChB programme.*

**Section 1: Demographics**

**1. Please select the health board in which you are currently based from the following options:**

- NHS Grampian
- NHS Highland
- NHS Shetland
- NHS Orkney
- Other: Please state

**2. Please select whether you are currently based in primary or secondary care:**

- Primary care
- Secondary care

**4. Please select your grade from the following options;**

- Year Four Medical Student
- Final Year Medical Student
- FY1
- FY2
- Junior Clinical Fellows
- CT/IMT/GPST/ST1-2
- ST3+/Senior Clinical Fellows
- GP
- SAS
- Consultant
- Other – define

**5. Have you ever had teaching on the topic of ionising radiation? Select which apply**

- Lectures
- Tutorials
- Online e-learning module
- None

**Section 2: Self-Rating Scales of Knowledge and Importance**

**6. Please rate your knowledge of the radiation risks of common radiological investigations on the following scale:**

| 1 | 2 | 3 | 4 | 5 | 6 | 7 |
| --- | --- | --- | --- | --- | --- | --- |
| None at all | Poor | Limited | Average | Fairly Good | Good | Very Good |
|  |  |  |  |  |  |  |

**7. How important do you feel the knowledge of radiation risks applies to the role you are currently in?**

| 1 | 2 | 3 | 4 | 5 | 6 | 7 |
| --- | --- | --- | --- | --- | --- | --- |
| Not important at all | Not important | Slightly not important | Neutral | Slightly important | Important | Extremely important |

**Section 3: Knowledge Assessment.**

**Marks allocated as detailed below, to give a total mark out of 9.**

**8. Select which modalities would expose patients to ionising radiation from those listed below: Multichoice selection**

Ultrasound scan  - **Incorrect**

MRI scan  - **Incorrect**


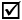

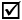
CT scan


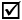
PET-CT


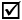
Chest radiograph (CXR)


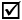
Mammogram

Angiogram

**0 to 1 correct – no marks**

**3 or more correct – 0.5 marks**

**5 correct – 1 mark**

9. What radiological investigation(s) do you request most often?

*The surrounding environment in the UK typically means that the background radiation for the average person is approximately 2.7mSv per year. This dose is equivalent to 135 plain posterioanterior chest x-rays (0.02mSv). Given this information, please answer the questions below.*

**9. What is the equivalent dose of radiation from a plain anterior-posterior abdominal radiograph when compared to a CXR?**

An AP abdominal radiograph is equivalent to ______ plain PA chest radiographs

1 **- Incorrect**


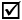
15 **- Incorrect**

35

50 **- Incorrect**

400 **- Incorrect**

Don’t know

**Incorrect/don’t know – 0 marks**

**Correct – 1 mark**

**10. What is the equivalent number of plain chest radiographs for each of the following investigations with regards to the radiation dose?**

**Part A: CT Head**

*A CT head is equivalent to ____ plain chest radiographs*

10 **- Incorrect**

20 **- Incorrect**


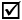
50 **- Incorrect**

100

400 **- Incorrect**

500 **- Incorrect**

Don’t know

**Incorrect/don’t know – 0 marks**

**Correct – 1 mark**

**Part B: CT Chest**

*A CT chest is equivalent to ____ plain chest radiographs*

10 **- Incorrect**

20 **- Incorrect**

50 **- Incorrect**


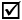
100 **- Incorrect**

400

500 **- Incorrect**

Don’t know

**Incorrect/don’t know – 0 marks**

**Correct – 1 mark**

**Part C: CT Abdomen & Pelvis**

*A CT abdomen & pelvis is equivalent to ____ plain chest radiographs*

10 **- Incorrect**

20 **- Incorrect**

50 **- Incorrect**

100 **- Incorrect**


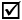
400 **- Incorrect**

500

 Don’t know

**Incorrect/don’t know – 0 marks**

**Correct – 1 mark**

**11. What is the additional lifetime risk of fatal and non-fatal cancer from following investigations:**

**Part A: CT Head**

*There is a 1 in ______ additional lifetime risk of fatal and non-fatal cancer following a CT head.*


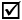


1:10,000

1:2500 **- Incorrect**

1:2000 **- Incorrect**

1:1000 **- Incorrect**

1:750 **- Incorrect**

No additional risk **- Incorrect**

Don’t know

**Incorrect/don’t know – 0 marks**

**Correct – 1 mark**

**Part B: CT Chest**

*There is a 1 in ______ additional lifetime risk of fatal and non-fatal cancer following a CT chest.*


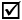
1:10000 **- Incorrect**

1: 2500

1:2000 **- Incorrect**

1:1000 **- Incorrect**

1:750 **- Incorrect**

No additional risk **- Incorrect**

Don’t know

**Incorrect/don’t know – 0 marks**

**Correct – 1 mark**

**Part C: CT Abdomen & Pelvis**

*There is a 1 in ______ additional lifetime risk of fatal and non-fatal cancer following a CT abdomen and pelvis.*

1:10000 **- Incorrect**


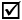
1:2500 **- Incorrect**

1:2000

1:1000 **- Incorrect**

1:750 **- Incorrect**

No additional risk **– Incorrect**

Don’t know

**Incorrect/don’t know – 0 marks**

**Correct – 1 mark**

**12. Which groups of patients carry a higher risk/are more sensitive to the risks of radiation? Multichoice selection**


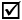


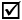
Children

Female 16-60

Male 16-60 **- Incorrect**

Elderly **- Incorrect**

Equal risks in each patient group **- Incorrect**

Don’t know **- Incorrect**

**0 correct – no marks**

**1 correct – 0.5 marks**

**2 correct – 1 mark**

**Section 4: Additional Information**

**13. Do you think more teaching on this topic would be beneficial?**

- Yes
- No
- Don’t know

**14. What type of learning package would you prefer to use to improve knowledge?**

- Lecture
- Tutorial
- Online e-learning module
- Other -

**15. Any further comments:**

## *8.3 Appendix 3: Further Comments*

Answers from the last question of the survey: “Please outline any further comments” are displayed below.

| 1 | I was surprised at how little confidence I had answering these questions and I feel like we should be taught more about the risks of these investigations at university. Maybe teaching would be more effective in 5th year as we get closer to actually ordering these investigations. |
| --- | --- |
| 2 | Interesting topic! I would say that as a GP trainee I think more about radiation and risks as have to 'sell' the referral to the radiologist (even for x rays) whereas my experience in hospital is that most would get a chest x Ray as a baseline investigation at hospital. |
| 3 | My knowledge on this may (hopefully) be better than my peers as my wife is a radiographer |
| 4 | - |
| 5 | Thank you and good luck! |
| 6 | An awareness is important but full on training is probably not necessary unless your specialty uses one mode particularly often. |
| 7 | Think further teaching comparing relative risks of different investigations could be useful |
| 8 | We expect the specialists to advise us. |
| 9 | This has highlighted a knowledge gap regarding radiation exposure. A short lecture / tutorial would be really helpful on this as it would be good to communicate with patients when discussing scans. |
| 10 | As an FY I remember having some teaching on this with similar risks but I found this difficult so haven’t remembered very well - therefore maybe annual teaching / pop up reminders on the system when you order investigations (if online requests) or poster reminders in doctor’s offices would be useful to remind doctors of the exposure risks! |
| 11 | . |
| 12 | My understanding is that the scale of risk or its existence at all is controversial and not certain. Interested to know more about the current evidence and guidance. |
| 13 | Unsure if I was supossed to guess the relative risks, but I've just said don't know which is a correct answer! |
| 14 | N/A |
| 15 | I work in Old Age medicine - generally lifetime risks are less relevant at this stage! We do consider other risks of investigation though, such as risks of overinvestigation and incidental findings |
| 16 | Although I think it would be helpful to have a concrete number to refer to that quantifies the risk of radiation, I do not think it would significantly change the number of investigations I request - I already would reduce exposure where possible knowing the general tennant - if radiation can be avoided then avoid it. For example knowing the CT head life time risk of additional exposure will not change my practice in terms of hyperacute stroke assessment - a CT head is required regardless of this risk. Where logistically possible and not felt to delay appropriate treatment we have organised MRI instead (patients under18). |
| 17 | - |
| 18 | I think it is important that certain professionals (radiographers/radiologists) are custodians of the specific knowledge relating to the tests that are performed. Whilst having some knowledge of ionising radiation risks in medical practice is important, the level of specific detail that is indicated by the questions above seems more than is appropriate for most practicing clinicians. |
| 19 | The problem is always balanced decision making. The issue of a 1/2500 cancer is not a priority when dealing with situations that have a 25% 30 day mortality risk. You need to have significant thought about patient information in decision making, patients are frequently subject to ionising radiation with zero consent, zero knowledge of the risks and decisions made by junior medical staff who have no experience or ability to consider broader clinical risks. I think the most beneficial outcome would be have patient information leaflets on the wards explaining these risks for patients rather than purely focusing on medical staff knowledge as that's not a 21st good medical practice approach! |
| 20 | i don't know the answers to many of the questions, but the learning I have absorbed is that there is always a risk with radiation and the risk to pt needs to be weighted up against the information that may be gleaned from imaging and how it will affect treatment. |
| 21 | None |
| 22 | Difficult to answer some of these, various studies report different radiation doses and lifetime risk. Risk and exposure depends on practitioner, appropriate collimation, distance and technique (including average attempts to obtain image). |
| 23 | well done for researching this topic |
| 24 | The default of surgical teams to get CT scans for ED patients needs to be addressed - what happened to appendicitis being a clinical diagnosis? The defensive medical world that we live in means that the number of CT heads done in ED under NICE guidelines is ridiculous. |
| 25 | Probably should be more emphasized on statutory learning modules/deanery teaching. Delivered teaching would be ideal. |
| 26 | out of date with this but aware modern CT scanning carries much less radiation than previously |
